# Divergent Effects of Beliefs in Heaven and Hell on National Crime Rates

**DOI:** 10.1371/journal.pone.0039048

**Published:** 2012-06-18

**Authors:** Azim F. Shariff, Mijke Rhemtulla

**Affiliations:** 1 Department of Psychology, University of Oregon, Eugene, Oregon, United States of America; 2 Center for Research Methods and Data Analysis, University of Kansas, Lawrence, Kansas, United States of America; Ecole Normale Supérieure, France

## Abstract

Though religion has been shown to have generally positive effects on normative ‘prosocial’ behavior, recent laboratory research suggests that these effects may be driven primarily by supernatural punishment. Supernatural benevolence, on the other hand, may actually be associated with less prosocial behavior. Here, we investigate these effects at the societal level, showing that the proportion of people who believe in hell negatively predicts national crime rates whereas belief in heaven predicts higher crime rates. These effects remain after accounting for a host of covariates, and ultimately prove stronger predictors of national crime rates than economic variables such as GDP and income inequality. Expanding on laboratory research on religious prosociality, this is the first study to tie religious beliefs to large-scale cross-national trends in pro- and anti-social behavior.

## Introduction

A growing program of research from across the social sciences now supports the long-held claim that religion positively affects normative behavior (see [1] for a review). Religiosity shows consistent positive correlations with charity and volunteerism [2], and negative relations with lax attitudes about the justifiability of moral transgressions [3]. Moreover, experimental work has shown that religious priming increases ‘prosocial’ generosity and cooperation, and decreases cheating [4]–[6].

However, recent studies suggest that not all religious beliefs are equal in this respect. Though supernatural punishment is associated with increases in normative behavior, laboratory research reveals the concept of supernatural benevolence to be associated with *decreases* in normative behavior. For example, university students with stronger beliefs in in God’s punitive and angry nature tended to be the least likely to cheat on an academic task, whereas stronger beliefs in God’s comforting and forgiving nature significantly predicted higher levels of cheating [7]. These results remained robust after controlling for plausible third variable candidates.

This pattern of results is consistent with theories highlighting the effectiveness of supernatural punishment–specifically–at regulating moral behavior and, as a result, group cooperation [8]–[9], [1]. These theories argue that human punishment is a highly effective deterrent to anti-social behavior within groups, but one that faces inevitable limitations of scale. Human monitors cannot see all transgressions, human judgers cannot adjudicate with perfect precision, and human punishers are neither able to apprehend every transgressor, nor escape the potential dangers of retribution. Divine punishment, on the other hand, has emerged as a cultural tool to overcome a number of those limitations. Unlike humans, divine punishers can be omniscient, omnipotent, infallible, and untouchable-and therefore able to effectively deter transgressors who may for whatever reason be undeterred by earthly policing systems.

Supernatural benevolence, however, is not theorized to be similarly effective at stabilizing cooperation within groups. Moreover, the evidence thus far suggests that though the more ‘positive’ religious attributes may provide their own benefits, such as better self-esteem [10] or health coping [11], their role in encouraging moral behavior may be, at best, minimal and, at worst, negative.

Indeed, recent social psychological research has used priming experiments to establish causality in this negative relationship. Christian participants spent ten minutes writing about God’s forgiving nature, God’s punitive nature, a forgiving human, a punitive human, or a neutral control. In a subsequent and purportedly unrelated task, participants were given the opportunity to overpay themselves for the study using a common measure of petty theft where participants ostensibly privately assign themselves payment for correctly answered anagrams. Though, on average, participants in the punishing God and both human conditions overpaid themselves less than 50 cents more than what they deserved for their anagrams, and did not statistically differ from the neutral condition, those who wrote about a forgiving God overpaid themselves significantly more-nearly two dollars (Unfortunately, because the level for both the control and the punishing god prime conditions were at a statistical floor in this study, the ability of punishing god primes to reduce levels of theft could not be reliably assessed) [12].

The important question that arises from all of these religion and prosocial behavior studies is to what degree do these laboratory-based effects translate to large-scale societal effects? Ultimately, what is the relation between the punitive and benevolent aspects of religion and pro/anti-social behavior in the world beyond the laboratory? If the theories and findings described above reflect substantial psychological effects, then differences in supernatural punishment and benevolence beliefs should also differentially predict transgressive actions such as criminal behavior at the societal level. Using large international datasets, we investigated this prediction by examining the role of beliefs in heaven and hell.

## Materials and Methods

Data for belief in hell, belief in heaven, belief in God, and religious attendance were taken from the 1981–1984, 1990–1993, 1994–1999, 1999–2004, and 2005–2007 waves of the World Values Surveys (WVS) and European Value Surveys [13]. Some countries included the question in multiple survey years (individual survey participants only participated once); others included the question at only one data collection wave. In total, these data were based on participants from 67 countries (*N* = 143,197; mean *N* per country = 2137, range = 362−9016). Weighted means were computed for each country based on a proportional weighting variable supplied with the WVS.

Belief in heaven, hell and God was assessed with the oral question, “Which, if any, of the following do you believe in?”, followed by a list of concepts including “Heaven,” “Hell” and “God”. Accepted answers were Yes and No. Religious attendance was assessed with the question, “Apart from weddings, funerals, and christenings, about how often do you attend religious services these days?”; the response options were 1 = More than once a week, 2 = Once a week, 3 = Once a month, 4 = Only on special holy days/Christmas/Easter, 5 = Other specific holy days, 6 = Once a year, 7 = Less than once a year, 8 = Never or practically never. A weighted average was computed for each country across all available data, using the supplied individual weighting variable.

Mean standardized crime rates were computed from the 10 crimes for which the United Nations Office on Drugs and Crime (UNODC) [14] had reliable statistics: homicide (*N* = 67 countries), robbery (*N* = 51), rape (*N* = 48), kidnapping (*N* = 46), assault (*N* = 48), theft (*N* = 47), drug crime (*N* = 47), auto theft (*N* = 28), burglary (*N* = 43), and human trafficking (*N* = 39). These data were compiled by the UNODC from national government sources, including police and court records, national statistics ministries, and other national government bodies. For each individual crime, the annual data from 2003–2008 were averaged to form a single 5-year measure, except for homicide data, which were taken from the latest available single year (range 2004–2010).

Per capita GDP data were taken from the 2007 CIA World Factbook [15]. Dominant religion, Gini coefficient data and life expectancy data were 2011 estimates (or the latest available estimate where 2011 estimates were not available) drawn from the CIA World Factbook [16]. Urban density data reflect the percentage of each country’s population residing in urban areas in 2005; these data are from the United Nations Department of Economic and Social Affairs [17]. Population imprisonment data were taken from the World Prison Population List [18]. Personality data were taken from Schmitt, Allik, McCrae & Benet-Martínez [19], who administered translated versions of the Big Five Inventory of personality differences to 17,837 individuals in 56 nations. We elected to add only conscientiousness, agreeableness and neuroticism to the analysis as they have been previously linked to pro- and anti-social behavior, however, results do not dramatically change if all five personality factors are entered into the regression. All the above data are publicly available.

All variables were entered into a series of linear regression equations, with each crime regressed on beliefs in heaven and hell as well as all covariates. Standardized beta coefficients for each of these analyses, as well as for the aggregate average of the 10 crimes are presented in Table 1.

**Table 1:** Regression of individual crime rates on beliefs in heaven, hell, and 13 covariates.

		Assault	Motor	Burglary	Drug	Homicide	Human Traffick-ing	Kidnapping	Rape	Robbery	Theft	Average of All Crimes
Analysis with no covariates												
	**Belief in heaven**	1.728***	1.905***	1.536***	1.25***	1.244***	–0.196	0.325	1.733***	1.29***	1.325***	1.958***
	**Belief in hell**	–1.788***	–2.186***	–1.876***	–1.594***	–0.947***	0.337	–0.174	–1.79***	–1.288***	–1.779***	–1.941***
Analysis including covariates												
	**Belief in heaven**	2.079***	1.094*	0.931	1.314**	0.935*	–0.370	–0.843	2.031***	0.280	1.020***	1.820***
	**Belief in hell**	–2.075***	–0.979	–0.853	–1.413**	–0.850*	0.725	1.175*	–2.075***	–0.376	–1.048**	–1.698***
	Roman Catholic	–0.258	0.085	0.307	0.157	0.140	0.090	0.242	–0.228	0.283	0.085	0.112
	Other Christian	0.069	–0.005	0.351	0.415*	–0.044	–0.049	0.091	0.146	–0.099	0.297**	0.170
	Muslim	0.106	0.021	0.127	0.455*	–0.134	–0.477*	–0.114	0.137	–0.136	0.077	0.051
	Religious attendance	–0.444	0.361	0.310	0.055	–0.247	0.972***	0.864*	–0.509	0.478	0.293*	–0.124
	Pop. imprisoned	–0.031	0.369***	−0.071	−0.051	0.213	−0.158	−0.161	0.137	0.047	−0.048	0.075
	Gini coefficient	−0.132	−0.451*	−0.080	0.029	0.299	0.018	0.077	−0.048	0.206	0.399***	0.185
	GDP per capita	−0.079	0.022	0.394	0.785***	−0.249	−0.519*	0.324	0.446*	−0.288	0.345***	0.053
	Life expectancy	−0.176	0.142	−0.286	−0.328	0.060	0.624*	−0.167	−0.561*	0.115	−0.183	0.083
	Urbanicity	0.419	0.062	0.057	−0.057	−0.119	−0.394	−0.141	0.255	0.135	0.002	−0.137
	Belief in God	−0.513*	−0.045	−0.027	0.155	−0.261	0.344	0.555	−0.199	0.171	−0.322*	−0.475*
	Conscientiousness	0.434	0.160	−0.234	−0.511*	0.081	0.307	0.137	−0.320	0.489*	−0.272*	0.400
	Neuroticism	0.081	0.270	−0.091	−0.440*	−0.116	0.218	0.104	−0.244	0.390*	−0.321*	0.095
	Agreeableness	−0.069	0.163	0.174	0.096	0.039	−0.181	0.139	0.027	−0.069	0.546***	0.012

Note. As recommended by [22], regression results are presented with and without covariates. Asterisks indicate significance levels according to Wald tests (^*^  =  significant at α = .05, ^**^  =  significant α = .005, ^***^  =  significant at α = .001). Significance tests should be interpreted cautiously, as no correction has been made for inflated error rates due to performing a large set of analyses. As 12 analyses were performed, only those effects that are significant at α = .005 or below may be confidently interpreted as significant.

## Results

As predicted, rates of belief in heaven and hell had significant, unique, and opposing effects on crime rates. Belief in hell predicted lower crime rates, 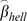 = −1.941, *p*<.001; whereas belief in heaven predicted *higher* crime rates, 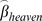 = 1.958, *p*<.001 (Note that these are standardized regression coefficients, so they may be interpreted as effect sizes). Controlling for the effect of belief in heaven, a 1 SD increase in belief in hell resulted in an almost 2 SD decrease in national crime rate; conversely, controlling for the effect of hell, a 1 SD increase in belief in heaven resulted in an almost 2 SD *increase* in national crime rate. Analyzing each crime individually revealed the same significant pattern of effects for 8 of the 10 individual crimes (kidnapping and human trafficking excepted; see Table 1).

To discount the role of obvious third variables, we conducted a second analysis with several covariates. Dominant religion was included in the form of three dummy coded variables that indicated a country’s predominant religious group as Roman Catholic, Other Christian, and Muslim [16]. We included two standard economic factors relevant to crime rates: income inequality (measured by the Gini coefficient, [14] and GDP per capita [15]; national imprisonment rates as a measure of a country’s punitive nature [18]; two demographic factors that reflect important differences between nations: life expectancy [16] and urban density [17]; three of the “Big Five” personality variables that have been previously tied to pro- and anti-social behavior: conscientiousness, neuroticism and agreeableness [19]; and finally two factors specifically focused on the religiousness of the different nations: belief in God and religious attendance [13] (Reported results use maximum likelihood estimation to deal with missing data. Listwise deletion (N = 53) gave the same pattern of significant results).

Despite many of these variables–especially poverty and income inequality–being frequently discussed as determinants of crime [20], [21], only belief in God had a significant effect on average crime rates over and above the effects of belief in heaven and hell, which remained highly significant (both *p*s <.001). Moreover, when analyzing the individual crimes with these covariates, beliefs in heaven and hell still emerged as the strongest predictors for 5 of the 10 crimes (see Table 1).

**Figure 1:**
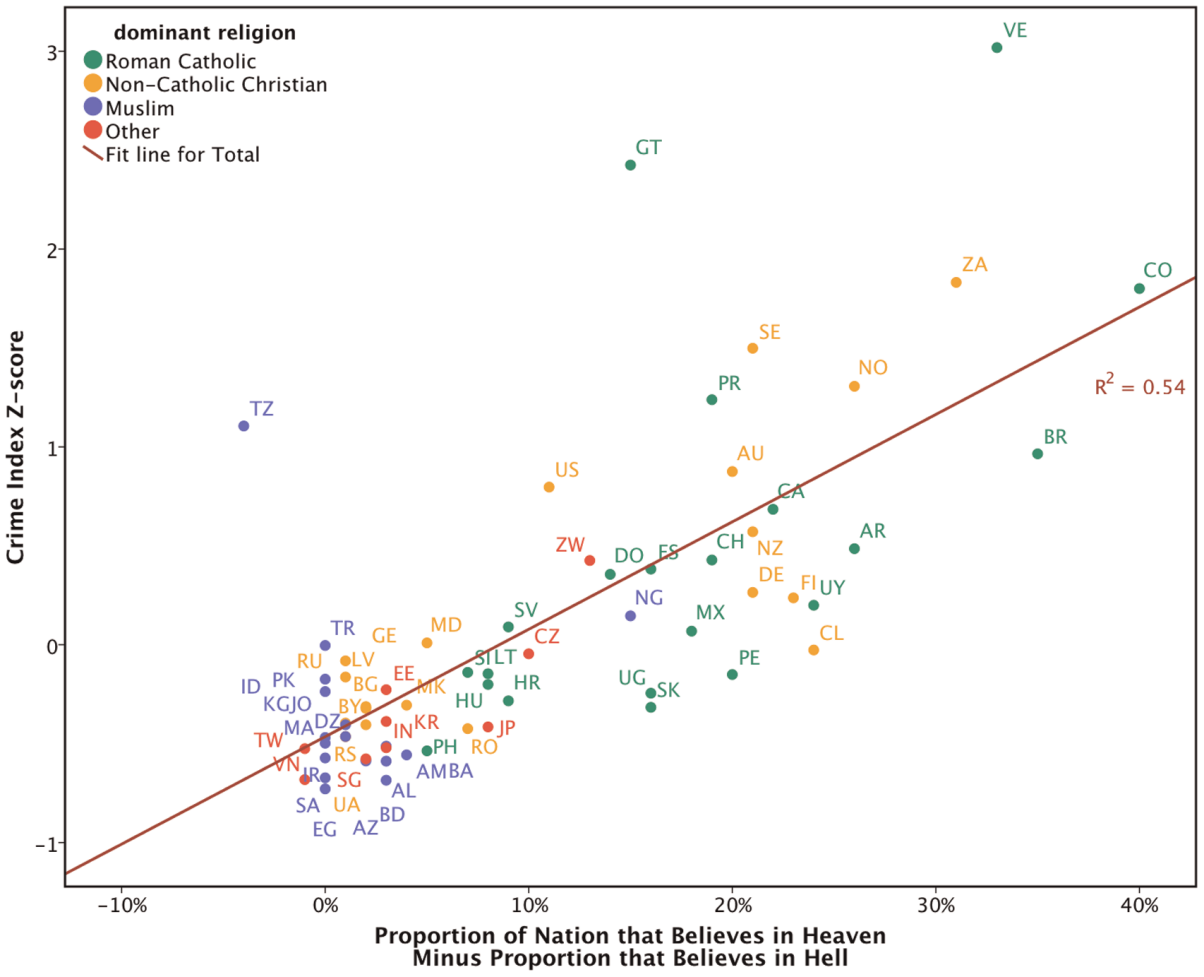
Crime rate z-scores as a function of how much higher the proportion of a nation that believes in heaven is compared to the proportion that believes in hell. R^2^ = .54.

The strength of these effects is made clear by examining crime rates as a function of the degree to which a greater percent of people in a nation believe in heaven than in hell. As Figure 1 depicts, rates of belief in heaven are virtually always higher than rates of belief in hell; however, as the degree of that discrepancy increases (from roughly equal proportions of the population believing in the two concepts, to up to 40% more believing in heaven than in hell), so too do crime rates.

The pattern of results is robust to spatial and cultural variability. The same pattern of results emerges in three out of the four continental zones for which there were sufficient data-namely, Africa, South and Central America, and Europe plus Canada, USA, Australia and New Zealand. The same pattern also emerges for three out of the four religious groups that form national majorities–predominantly Roman Catholic, predominantly non-Catholic Christian, and predominantly ‘Other,’ which comprises of either unaffiliated majorities, or more localized majority religions such as Hinduism, Shintoism and syncretic religions that combine Islam and Christianity with traditional indigenous religions (see Figure 1). The only exception to this observation is predominantly Muslim countries in Asia, for which the uniformly high levels of both belief in heaven and hell (*M*s = 93% and 91% respectively), produce insufficient variance for prediction.

## Discussion

The present analysis has uncovered two strong, unique, and reliable relations between religious belief and national crime rates. The degree to which a country’s rate of belief in heaven outstrips its rate of belief in hell significantly predicts higher national crime rates. Statistically, this finding manifests in two independent effects: the strong negative effect of rates of belief in hell on crime, and the strong positive effect of rates of belief in heaven on crime. Both of these effects follow from predictions based on recent laboratory findings [7], [12] and on theories that ascribe socio-cultural functions of religions [1]. Indeed, these findings coalesce with theoretical and empirical work suggesting that beliefs in punishing and omniscient supernatural agents spread across historical societies primarily because of their ability to foster cooperation and suppress anti-social behavior among anonymous strangers.

### Limitations and Future Directions

First and foremost, these findings are correlational, and thus reverse-causation and third variable explanations need to be discounted before causal claims can be firmly endorsed. However, at least two reasons suggest that a causal effect of these religious beliefs on crime is a plausible explanation for the pattern of results.

First, obvious third variable candidates such as differences between countries in national personality, wealth, wealth distribution, and general religiosity show no indication of driving the effects. Second, numerous lab studies have established direct causal effects for religious beliefs on both pro- and anti-social behaviors. The possibility remains that the lab effects and the international crime rate effects are entirely unrelated, but parsimony suggests that both are, at least to some degree, a reflection of the same underlying causal story. Nevertheless, future research would be beneficially directed towards addressing possible alternative explanations.

Understanding the mechanisms underlying these effects is another important area of future inquiry. As discussed in the introduction, much research now supports the conclusion that the *pro*social effects of religion are due, at least in part, to the fear of supernatural punishment serving as a deterrent to transgressive behaviors. The *anti*social effects of religious benevolence, which, counting the current findings, have been now documented in several studies (e.g. [7], [12]), are, however, less well understood. Thus far, researchers have focused their speculation on the idea that divine forgiveness offers individuals a way to cleanse their moral palate, and thereby feel more licensed to transgress again. That is, divine forgiveness, like its earthly variant, may act as a counter-deterrent. This hypothesis garners support from several related studies (e.g. [23], [24]), but remains open to further testing.

Finally, in the efforts to uncover the mechanisms at play, it will be important to examine these real-world effects at the level of the individual. The present findings tie rates of belief at the societal level to national crime rates; the direct causal explanation for this effect is that individuals who believe in heaven and not hell take punishment less seriously and are thus more likely to commit crimes. It is also possible, however, that an intervening variable or variables are at work at the societal level. It may be that widespread belief in a forgiving god may lead a society to value forgiveness over punishment, and that this secular value in turn affects crime rates. In this scenario, an individual’s belief in heaven or hell may not directly affect her proclivity to engage in criminal behavior. The direct causal explanation is most closely in line with the experimental findings, but it could well be that both the direct and indirect mechanisms are at work. To assess individual-level effects simultaneously with societal-level effects, it will be necessary to collect data with both national crime rates and individual tendencies toward immoral behavior.

### Conclusions

These findings not only help to explain the differential relations that supernatural punishment and benevolence have to moral behavior-a topic of considerable recent interest in the social sciences-but also raise important questions about the potential impact of religious beliefs on global crime. Though little research in economic and social policy concentrates on religion, economists have observed that hell beliefs may positively impact the economic growth of developing nations [25]. It is quite possible that the present findings, which tie belief in hell to lower levels of anti-social behavior, may serve as one of the key explanatory mechanisms underlying this economic trend. Indeed, simply given the strength of the results compared to standard economic indicators such as GDP and the Gini coefficient, social scientists and policy makers might more deeply consider the cultural impact of religious beliefs in future work.
